# Musculoskeletal health and work ability in physically demanding occupations: study protocol for a prospective field study on construction and health care workers

**DOI:** 10.1186/1471-2458-14-1075

**Published:** 2014-10-16

**Authors:** Lars-Kristian Lunde, Markus Koch, Stein Knardahl, Morten Wærsted, Svend Erik Mathiassen, Mikael Forsman, Andreas Holtermann, Kaj Bo Veiersted

**Affiliations:** National Institute of Occupational Health, Gydas vei 8, 0336 Oslo, Norway; Department of Occupational and Public Health Sciences, Centre for Musculoskeletal Research, University of Gävle, Kungsbäcksvägen 47, Gävle, Sweden; Institute of Environmental Medicine, Karolinska Institutet, Stockholm, Sweden; The National Research Centre for the Working Environment, Lersø Parkallé 105, Copenhagen, Denmark

**Keywords:** Physical exposures, Work ability, Musculoskeletal disorders, Accelerometer, Heart rate monitoring, Electromyography, Ground reaction force

## Abstract

**Background:**

Musculoskeletal disorders have a profound impact on individual health, sickness absence and early retirement, particularly in physically demanding occupations. Demographics are changing in the developed countries, towards increasing proportions of senior workers. These senior workers may have particular difficulties coping with physically demanding occupations while maintaining good health.

Previous studies investigating the relationship between physical work demands and musculoskeletal disorders are mainly based on self-reported exposures and lack a prospective design. The aim of this paper is to describe the background and methods and discuss challenges for a field study examining physical demands in construction and health care work and their prospective associations with musculoskeletal disorders, work ability and sickness absence.

**Methods and design:**

This protocol describes a prospective cohort study on 1200 construction and health care workers. Participants will answer a baseline questionnaire concerning musculoskeletal complaints, general health, psychosocial and organizational factors at work, work demands, work ability and physical activity during leisure. A shorter questionnaire will be answered every 6^th^ months for a total of two years, together with continuous sickness absence monitoring during this period. Analysis will prospectively consider associations between self-reported physical demands and musculoskeletal disorders, work ability and sickness absence. To obtain objective data on physical exposures, technical measurements will be collected from two subgroups of N = 300 (Group A) and N = 160 (Group B) during work and leisure. Both group A and B will be given a physical health examination, be tested for physical capacity and physical activity will be measured for four days. Additionally, muscle activity, ground reaction force, body positions and physical activity will be examined during one workday for Group B. Analysis of associations between objectively measured exposure data and the outcomes described above will be done separately for these subpopulations.

**Discussion:**

The field study will at baseline produce objectively measured data on physical demands in the construction and health care occupations. In combination with clinical measurements and questionnaire data during follow-up, this will provide a solid foundation to prospectively investigate relationships between physical demands at work and development of musculoskeletal disorders, work ability and sickness absence.

## Background

Musculoskeletal disorders (MSD) are a major problem in the European population, with a prevalence of over 100 million people reporting discomfort in muscles or joints [1]. Studies have shown that decreased musculoskeletal function will negatively affect work ability [2, 3], and that MSD is one of the major reasons for sickness absence from work [1]. Consequently, MSD leads to major economic consequences at a society level due to their negative effects on sickness absence, work ability and early retirement [1, 4].

Risk factors for developing MSD are present both during work and leisure. Individual factors such as age, genetics, lifestyle, beliefs and general health have shown to be important modifiers of risk [5–8]. Moreover, both physical, social, organizational and psychological factors at work are known to be risk factors for MSD [9]. Physical demands at work are considered a crucial determinant for developing MSD, including frequently stated occupational risk factors such as high muscular loads, working and lifting in stooped, restricted or twisted postures, repetitive movements, and working with elevated arms [5, 10, 11].

Construction and health care work are two sectors in working life where the above-mentioned physically demanding risk factors occur to a high extent [12–15]. These occupations also show high prevalence of musculoskeletal pain in the neck, shoulder and low back [16–19].

Continuous and/or repeated force exertions across a significant period of time may result in tissue changes. Such changes can be positive (adaptation) or negative (reduced capacity). When tissues are exposed to repeated force exertions for several consecutive days they may develop a reduced tolerance for new exertions. A mismatch between work demands and individual performance capacity is likely to increase the risk of getting MSD, which, in turn may be associated with decreased capacity [20]. The result may then, if the job tasks remain unchanged, further increase the risk of MSD in a vicious circle. The term “work ability” is often used to describe this relationship between demand and capacity. Previous research has shown that high physical demands influence work ability in a negative manner and that reduced work ability may be a predictor of long term sickness absence and early retirement [21, 22].

Physical capacity, including aerobic capacity and muscular strength, will on average decrease with age, so senior workers (>50 yrs) may be particularly prone to develop insufficient work ability [23]. This could be a contributing explanation to age-related differences in work ability in physically demanding occupations, and to an increasing prevalence of MSD with seniority [24, 25]. This is an important issue, since the proportion of seniors in the work force is increasing, while, at the same time workers need to remain in the labor market up to a higher age, for instance driven by adjusted retirement rules [26].

While some studies have shown positive training effects from occupations with high physical demands [27, 28], other studies on seniors with a history of manual labor have associated a longer lifetime of physically demanding work with higher rates of disability, lowered physical function and reduction in muscular strength [29–31]. This has initiated a discussion of the positive or negative health effects of manual labor for senior workers [32, 33]. Paradoxically, physical activity during leisure time is regarded as important for maintaining and increasing physical capacity and work ability [22, 34, 35], while physically demanding work may be harmful. Disentangling this paradox requires access to data on physical activity patterns during both work and leisure.

Substantial research has been carried out on physical exposures in occupational settings, but scientific documentations obtained by objective measurement methods are rare [36]. Questionnaires, which have been the predominant instrument for collecting exposure information, cannot in detail capture the complexity of physical exposures at work, and their criterion validity may also be questioned, let alone they may give biased exposure data. For many exposures observational methods are also less accurate than technical measurements [37]. These problems encourage the use of objective methods for exposure measurement [38]. As pointed out by several systematic reviews, another major drawback of available epidemiologic research on musculoskeletal risk factors is, to a large extent, that it is based on cross-sectional designs, and thus cannot convincingly entangle causal relationships between exposure and outcome [39–45]. Also, relevant outcomes are known to vary across time in an individual, and a conventional registration of outcome once, after a certain follow-up time, may be too crude to reflect the characteristics of that outcome [46, 47]. In order to understand the genesis of MSD, longitudinal and frequent monitoring of both exposures and outcomes is necessary.

We intend to carry out a prospective study on the effects of physically demanding exposures on MSD, work ability and sickness absence among construction and healthcare workers, assessing exposure by questionnaires and objective field measurements of muscle activity, postures, physical activity and whole body load (ground reaction force). We will also collect data on psychosocial and organizational factors at work, as well as individual characteristics in order to understand their relationships with physical exposures. Recent exposure measurement technology allows us to monitor loads and muscle activity for full 8 hour work shifts, and to measure physical activity continuously for several days during both work and leisure. Combining these measurements allows a comprehensive assessment of exposure patterns across a long time range. This will give a sound basis of information to investigate the extent to which different aspects of physical exposure in construction and health care are associated with the outcomes MSD, work ability and sickness absence, as monitored prospectively for two years.

The present paper describes the study design and data collection methods in this planned study.

### Research questions

The superior research questions in the study are:

1. To which extent are different physically demanding exposures at work prospectively associated with risk for musculoskeletal disorders, reduced work ability and related sickness absence?
2. To which extent do other factors modify the possible association between physically demanding exposures during work and musculoskeletal disorders, reduced work ability and related sickness absence?

## Methods and design

This project is designed as a longitudinal cohort study. Baseline and follow-up measurements during a period of two years will be done to collect relevant data from work and leisure on workers in construction and health care. Data collection consists of objective measurements of muscle activity, ground reaction force, body positions and physical activity during work and leisure time, and a 2 year period of self-report on psychosocial and organizational factors, working postures and work load, physical activity and exercise, health, sickness and disorders, and work ability.

### Study population

The recruitment of subjects for this study will be done at three construction enterprises and a group of municipality nursing homes and home care units employing a total of approximately 2000 workers. All white and blue collar workers at each participating construction site, and all staff at the health care facilities will be invited to answer the baseline questionnaire in connection to a mandatory meeting at their participating site. During follow-up each participant is followed individually. Considering gender, a natural selection will lead to a higher proportion of men in the construction sector and of women in the health care sector. Based on previous experience we expect at least 1200 subjects out of the approximately 2000 invited, to accept filling in the baseline questionnaire. Based on answers in the baseline questionnaire on suitability (selection criteria, see below) and willingness, 300 (group A – light measurement set-up) of the 1200 will wear instruments for recording physical activity data 24-hour a day for four to five consecutive days. Another separate group of 160 among the 1200 will be recruited for physical activity measurement similar to group A, complemented with one full-shift measurement of additional physical exposure data at work on the first measurement day (group B – full measurement set-up). Selection of subjects for the technical measurements, i.e. group A and B, will be drawn at random from the sample agreeing to participate in this, balancing for job titles. All selection processes will be carried out using unidentifiable participation numbers.

### Selection criteria for participation

Subjects will only be included in the study if they have adequate skills in reading and writing Norwegian. Further, individuals will not be included in group A or B if they have known allergic reaction to plaster/tape/bandages or are pregnant. Individuals diagnosed with cardiovascular disease, have reduced general condition, suffer from considerable musculoskeletal pain on the test day, or are diagnosed with back or shoulder disorders contradicting physical capacity testing will not be subjected to tests they cannot perform.

### Data collection period

**Figure 1:**
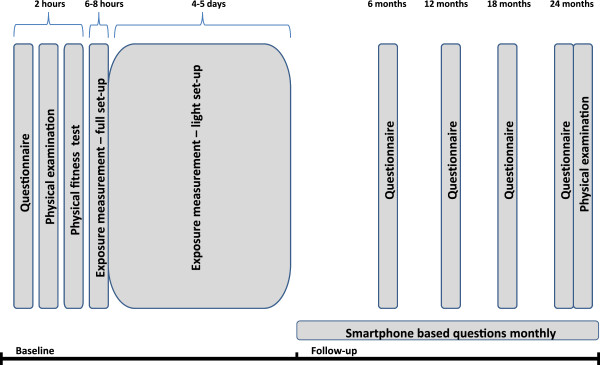
**Timeline for data collection.**

Data collection is planned to start in the 2^nd^ quarter of 2014 and end during the 4^th^ quarter of 2016. All baseline questionnaires will be collected by the 4^th^ quarter of 2014. With a weekly rate of 12 subjects in group A, and 8 in group B, all technical measures and thus all baseline data is estimated to be finished by the 1^st^ quarter of 2015. For each participant, the follow-up period will proceed for two years after baseline questionnaire is answered. The timeline of data collection is illustrated in Figure 1.

### Data collection procedure at baseline

The participating enterprises have agreed upon using scheduled mandatory meetings with all workers to give verbal and written information on the study and to distribute the baseline questionnaire. The enterprises have further given consent to allowing every participating worker up to two hours of paid time for testing and measurement set-up, and to have measurements being carried out during work shifts. Procedures for the different data collection methods are described below and summarized in Table 1.

**Table 1:** **Type of data collected within groups**

	All (n = 1200)	Group A (n = 300)	Group B (n = 160)
**Baseline**			
Questionnaire	x	x	x
Aerobic fitness		x	x
Muscular strength			x
Physical examination		x	x
Exposure measurement – light setup (4–5 days)		x	x
Exposure measurement – full setup (8 hours)			x
**Follow-up**			
Questionnaire 6, 12, 18 and 24 months	x	x	x
Sickness absence	x	x	x
Data collection by smartphone	x	x	x
Physical examination at 24 months		x	x

#### Questionnaire

Subjects will answer a self-administered baseline questionnaire with questions addressing personal and work related factors. The baseline questionnaire is constructed from validated questionnaires on psychosocial and organizational factors [48], working postures and work load [49–51], physical activity and exercise [52], health, sickness and disorders [53–56], and work ability [55, 57].

#### Aerobic fitness

Aerobic fitness will be determined using a cycle ergometer test (Ergometer 839 E, Varberg, Sweden) [58]. According to standard guidelines, subjects will perform 50 revolutions per minute at an external power between 75 and 150 watts. Heart rate will increase the first 2–3 minutes, then reaching a steady state. If heart rate is stable (±5 bpm) and greater than 120 beats per minute (bpm) between the 5^th^ and 6^th^ minute, the test will be terminated. If not, it will be prolonged. The mean steady-state heart rate (expectedly between the 5^th^ and 6^th^ minute) will be used to estimate maximal oxygen uptake (VO_2max_) based on the Åstrand monogram [59] modified for age and gender [60].

#### Muscular strength

Arm abduction strength will be tested sitting in a standardized position. A wire attatched to the floor via a hanging scale (Kern HBC HCN 100K200IP, KERN and SOHN GmbH, Balingen, Germany) is fixed to the upper arm, proximal to the lateral epicondyles on humerus [61]. The subject will get three attempts to achieve a maximal voluntary contraction (MVC), elevating the upper arm in 90° abduction in the scapular plane. Both arms are tested separately. Maximal strength in isometric back extension will be tested using a modified Biering-Sorensen test [62], applying a harness and a wire attached to the floor via a force transducer. The subject is urged to do three maximal back extensions pulling against the wired harness. Handgrip strength is tested for each arm separately using a hand dynamometer (Lafayette Instrument, Indiana, USA) according to standardized procedures [61]. For all tests, the maximal value of the three attempts obtained will be used. Maximum electromyography values (EMG_max_) for the upper Trapezius muscles and Erector Spinae (see below) will be determined during these tests.

#### Physical examination

In both group A and B a physical examination of the musculoskeletal system will be carried out by a physician or physiotherapist. A diagnostic instrument for upper-extremity musculoskeletal disorders will be used [63], as well as relevant clinical tests for low back and knee [64]. During the physical examination, data on blood pressure, height, body mass and waist circumference will also be collected.

#### Exposure measurements – light set-up

For group A, physical activity will be measured by five accelerometers and a heart rate monitor worn continuously for four to five consecutive days. The equipment will be mounted on a Monday morning and dismounted and returned on the following Friday. This will provide information on physical activity both during work and leisure.

#### Exposure measurements – full set-up

For group B, physical activity will be measured in a similar manner as in the light set-up*.* Accelerometers and heart rate monitor will be mounted on the participants on either Tuesday or Thursday morning and dismounted the following Monday or Tuesday, respectively. During work shift (8 hours) on the first day of physical activity measurement, participants in group B will in addition to physical activity measurements, carry equipment recording muscle activity, ground reaction force and postures. This additional equipment will be dismounted at the end of the work shift. *Observation* of participants work activities will be done during the entire work shift.

### Data collection procedure during follow-up

#### Questionnaire

A shortened version of the baseline questionnaire assessing the same issues will be sent to all participants six, 12 and 18 months after the baseline data collection. After 24 months, the participants will fill in a questionnaire similar to the one at baseline.

#### Sickness absence

Total long-term sickness absence stratified by diagnosis will be followed continuously in Norwegian registers (FD-Trygd). Change in employment status (professional title) will also be followed. Self-certified sick leave will not be registered. The labor and welfare service in Norway is responsible for safeguarding these registers and have approved use of these data. All participants are asked to give their consent to the use of register information.

#### Data collection by smartphone

Participants will receive a monthly text message for 24 months, containing an internet link to a small questionnaire based on validated questions on health complaints and working conditions (acute overexertion and working hours) the preceding month.

#### Physical examination at 24 months

After 24 months of follow-up, the physical examination will be repeated in group A and B.

### Instrumentation and application

#### Muscle activity

Muscle activity will be assessed using Electromyography (EMG) [65, 66]. Data will be collected by an 8-channel ambulatory system (Mobi 8, TSMi, Enschede, the Netherlands). This system has capacity to record raw EMG data at frequencies up to 2048 Hz, and a logger and battery capacity allowing data collection well above eight hours. The logger is small and light (115 × 98 × 37 mm, 165 g) and will be worn by the participant in a specialized light-weight sports vest during the work shift. Upper trapezius EMG will be collected using self-adhesive pre-gelled Ag/AgCl electrodes (Ambu Neuroline 720, Ambu, Ballerup, Copenhagen) centered 20 mm laterally from the center of the line from the acromion to the spine on vertebra C7 [65]. For measuring from the Erector Spinae (longissimus) muscles, electrodes will be placed two finger widths lateral to the processus spinosus of L1 [67]. A reference electrode will be placed at C5. For initialization of the logger and downloading of data the manufacturer’s software TMSi Polybench will be used. The procedures for assessing muscle activity from the trapezius and the low back is extensively documented [68].

#### Ground reaction force

Medilogic insoles sport (medilogic©insoles, T&T medilogic Medizintechnik GmbH, Berlin, Germany) sample at a rate of 300 Hz and record pressure between the foot and the shoe. Data will be recorded on a data logger (145 × 60 × 30 mm, 180 g) with eight hour measurement capacity worn by the participant. The manufacturer’s commercial software, Medilogic Software, will be used to start measurements and download data. An unpublished validation study evaluating the insoles under simulated field situations (standing, walking, lifting, catching an object and kneeling) showed that the system has a root-mean-square error ranging from 6.6 to 17.7% when comparing estimated forces to the “truth” registered by using a force plate reference system (AMTI LG6-4-1, Watertown, MA, USA). Kneeling may however lead to extreme bending of the insoles giving too high measurement error. Using time synchronized posture data and observational data such measurement periods will be removed from analysis.

#### Physical activity and postures

Actigraph GT3X + (Actigraph, Florida, U.S.A) is a triaxial accelerometer used to measure body positions and activity. The accelerometer is small (46 × 33 × 15 mm, 19 g), waterproof and have capacity to capture data at 30 Hz for up to 10 days continuously. The device is attached to the skin by double-sided tape, fixomull (BSN medical, Hamburg, Germany) and covered with transparent film (Tegaderm, 3 M, Minnesota, U.S.A). For the full exposure measurement set-up, a total of eight Actigraphs will be placed at the following bodily sites: upper back at the level of T1-T2, upper left and right arm 3 cm below the deltoid muscle insertion, at the top of iliac crest on the right side, medially between the iliac crest and the upper crest of patella on the right and left thigh, and on the lower right and left leg 3 cm below caput fibulae. The light exposure measurement will include the five accelerometers placed at the upper back, dominant upper arm, right hip, right thigh and right lower leg. Participants will be asked to wear the monitors at all times unless it leads to itching, rashes or to other discomforts, and receive additional tape, bandages, electrodes, and instructions on how to replace unintentionally detached devices. Initialization and downloading of data will be done using commercial software (Actilife version 5.5).

#### Physical activity - heart rate

Heart rate will be monitored using Actiheart (Camntech, Cambridge, United Kingdom), which is based on electrocardiography (ECG). Actiheart is a waterproof, compact and lightweight device designed to measure ECG Inter-Beat-Intervals and physical activity levels. The Actiheart can remain on the subject 24 hours a day and collect data for up to 72 h in total. The Actiheart software given by manufacturer will be used for set up, reading of data and charging the Actiheart. Recommended placements will be applied, attaching electrodes at the apex of the sternum and at the left intercostals at the level of the 6^th^ and 7^th^ costae [69]. The sensor has been found to be reliable and valid for use both in the laboratory [70] and free-living situations [71], and can be used as a measure for work ability [72].

### Data processing and analysis

For all mentioned modes of exposure, we will analyze data with a focus on retrieving variables describing the time patterns of exposure. Thus, we will quantify both levels (intensities), frequencies (change rates) and durations of relevant exposure modes.

#### EMG

The EMG signal recorded will be amplified, band-pass filtered 10–400 Hz [66], sampled at 1024 Hz, and stored as raw EMG. Following the recording, the raw EMG signal will be processed to eliminate ECG and powerline artifacts [73, 74]. To establish the EMG_max_ the highest 0.5 s (moving window) root-mean-square (RMS) value during the three attempts of a maximal voluntary contraction will be used. After normalization to EMG_max_, the EMG recordings will be processed to give the 10th (“static”), 50th (“median”) and 90th (“peak”) percentiles of the amplitude distribution [75]. Thereafter, using a discrimination level of 0.5 percent of EMG_max_ they will be processed to give the time-series of “activity” and “rest” sequences. First, the frequency of “EMG gaps” (episodes of muscle activity below the chosen discrimination level per minute) and “muscular rest” (proportion of total time with an EMG activity below the chosen discrimination level) will be examined for each of these three series. In addition, the number of uninterrupted episodes per hour with a muscle activity above the chosen discrimination level for a certain minimum of time (>1, 2.., 10 min), will be assessed, as well as the total relative duration of such episodes in the shift [76]. This approach is based on the analyses of sustained low level muscle activity episodes suggested by Østensvik and co-workers [77].

#### Ground reaction force

Following measurement, data sampled at 30 Hz will be exported using the manufactures software, Data logger reader (T&T medilogic Medizintechnik GmbH, Berlin, Germany). MATLAB R2013b (or later) will be used to continue data processing. The sum of pressure for each sole will be calculated and converted to a sum force value using the loaded area of the sole. This sum force is representing the vertical ground reaction force and will be normalized to participants’ body weight. Moving average filters will be used to smooth signal patterns. Discrimination levels will be set at 10%, 20% and 30% of body weight to reveal work exposures of increased force use. Frequency of exposures above discrimination levels as well as their total duration will be assed. The exposure will also be given in kilogram.

#### Accelerometer and heart rate

Using commercial and custom-made software [78, 79], Actigraph and Actiheart recordings will be processed to give body and arm positions, types of activity, heart rate during these activities, heart rate variability, number and frequency of steps, and estimates of energy expenditure. Duration and intensity of activity, inactivity, upper body inclination and arms above shoulder level will be of particular interest.

### Statistical analysis

The statistical analysis will address the two research questions stated earlier:

1. To which extent are different physically demanding exposures at work prospectively associated with risk for musculoskeletal disorders, reduced work ability and related sickness absence?
2. To which extent do other factors modify the possible association between physically demanding exposures during work and musculoskeletal disorders, reduced work ability and related sickness absence?

#### Research question 1

We will first use questionnaire data for all participants (N = 1200) to analyze the association between self-reported mechanical exposures and the outcomes musculoskeletal disorders in different body regions, work ability and register-based sickness absence. The same three outcomes will also be analyzed for their associations with the objectively measured physical activity and posture data (group A and B, N = 460), as well as EMG and force variables (group B, N = 160). All exposure modes will be first analyzed for its separate effect on outcome. Thereafter, analysis will carried out using several selected exposures effect on the outcome, thereby investigating their combined effect. For objective measures, exposure will be assumed time constant. We will produce both unadjusted and adjusted models. Potential confounders for the adjusted models, e.g. psychosocial factors or muscle strength, will be considered using an exploratory approach. The outcome variables will be assumed continuous, and we will have up till five observations per participant. For each outcome, and selected subsets of exposures, a linear mixed model approach will be used to model the association between exposures and outcome. The mixed model is robust to baseline differences and drop-out mechanisms that are missing at random.

#### Research question 2

Adjustment for possible confounders will be taken into consideration for the analysis described above. Some covariates can possibly act as moderators for the association between exposures and the outcome in question. Questionnaire data concerning individual, psychosocial and organizational factors, health and sickness, physical activity and exercise will be used to explore possible moderators. Objectively measured leisure time physical activity and physical capacity will also be included. The effect of a suspected moderator is modeled by allowing for an interaction between exposures and the moderating factor.

The statistical analysis will be done using both STATA version 13 (StataCorp, College Station, TX, USA) and IBM SPSS version 21 (IBM SPSS, New York, USA), or later versions.

### Study power

The sample size in this study is based on practical and economic considerations, and estimates are based on previous studies. From our collaborating enterprises we will have the possibility to approach approximately 2000 workers, as previously stated we expect at least 1200 to respond to the initial questionnaire. We will here address the question of statistical power for our sample size. For questionnaire data on outcome and covariates we will obtain N = 1200. When using questionnaire data to answer the two research questions, the two study populations, construction and health workers, will be analyzed both as one group and separately, giving a sample size of either N = 1200 for the whole population or N = 600 for each group. Based on previous experience we include in the power analysis a drop-out from baseline to first follow-up of 20%, and then 30% at the remaining follow-ups.

To investigate the power of the study design, we focus on research question 1 and 2, by using questionnaire data for both exposure and outcome assessment. We consider the exposure question «How physically demanding do you normally perceive your working situation?», and the outcome « Symptoms and complaints the last 4 weeks – lower back pain (intensity) ». The exposure is on a scale from 0 (nothing at all) to 12 (very very hard). The exposure distribution is based upon exposure data from Jebens et al. [80]. The outcome (intensity of pains) is rated on a four-point scale and may take the values 0 (not troubled), 1 (a little troubled), 2 (quite troubled) and 3 (seriously troubled). Based on cross-sectional data from the above mentioned study we assume a between-worker standard deviation (SD) of 0.68. The within-worker SD was assumed to be half the size of the between-worker SD (0.34). Data were simulated and estimated using a mixed model with random intercepts for subjects. The fixed effects included in the model depended upon the research question.

#### Research question 1

We wanted to examine the statistical power to obtain a significant association between the exposure and outcome variable for a group of 600 construction or health care workers. The exposure variable was included as fixed effect. When the exposure is low (exposure =1, very easy work), the average intensity of low back pain is assumed to be 0.5. The effect size was defined as the average increase in pain/symptoms when exposure increases from low (1 = very easy work) to high (9 = very hard work). The power of getting a significant association between exposure and pain/symptoms was estimated to be 0.80, 0.99 and 1.00 when the effect size is 0.1, 0.15 and 0.20, respectively.

#### Research question 2

To examine statistical power for research question 2, we introduced a moderation of the association between exposure and outcome by physical capacity, dichotomized into two categories of low and high physical capacity. The effect size was defined as for research question 1, but now allowed for a moderator effect of physical capacity. The effect of the exposure on the outcome is assumed to be half for workers with high physical capacity compared to workers with low physical capacity. When the exposure is low, the average difference in intensity of lower back pain between fit and unfit workers is assumed to be 0.5.

As fixed effects, we included physical capacity, exposure and an interaction term between exposure and physical capacity. With N = 600, the power of getting a significant result for a moderator effect for the physical capacity variable was estimated to be 0.60, 0.81, 0.93 and 0.98 when the effect size for physically fit workers is 0.15, 0.20, 0.25 and 0.30 respectively.

### Selection and missing data

To secure same information is given to all potential participants, all employees at participating work sites will be invited to a mandatory information meeting concerning the project. Questions towards the project will be answered directly by researchers giving the information. The importance of participation is further emphasized by both company leaders and researchers. Doing this we aim to minimize difference between the group who participate in the questionnaire part of the study and those declining the invitation. For the group participating in objective exposure measurements it will further be underlined that time employees use for physical examination and technical measurements is covered by the enterprises and will not affect the individuals’ paycheck or work conditions. In addition, we will offer participants to choose from both female and male study personnel to carry out physical examination and technical measurement preparations. Based on earlier experience there will also be focus on encouraging health care workers doing evening and night shifts to volunteer for technical measurements. Such efforts should increase the generalizability of the study. A certain initial decline of invitation and drop-out during follow-up must be expected. Based on previous experience we expect at least 1200 out of the total 2000 to answer baseline questionnaire and from these a drop-out from baseline to first follow-up of 20%, and for the remaining follow-ups 30% loss is estimated. We assume that the drop out will be larger in the population only answering questionnaire data than for the population subjected to direct exposure measurement, since the latter will be more closely approached. In case of missing covariates, due to missing questionnaire data, multiple imputation will be used [81]. Outcome will be censored when people leave work due to unemployment, rehabilitate pension or disability pension. However, based on the recent labor force survey in Norway [82], we do not expect that this will happen to any substantial proportion of study participants.

### Ethical aspects

This study was approved by the Regional Committee for Medical and Health Research Ethics in Norway (2014/138/REK sør-øst D). All subjects will be given written information on the purpose and methods in the study and will need to sign a written consent prior to participation.

## Discussion

This longitudinal cohort study will provide both self-reported and objectively measured data on physical exposures and physical activity during work and leisure, analyzed by methods reflecting both average exposure levels and exposure variation. This extensive exposure data will be analyzed for possible associations with MSD, work ability and sickness absence. This may provide knowledge of which factors that are of importance concerning the outcomes and thus indicate areas of focus within physically demanding occupations.

### Study population

In this study we include construction and health care workers because these workers represent occupations assumed to be characterized by heavy physical work. They are amongst the trades that employ the largest amount of workers in Norway and additionally, at the high end of reporting MSD [49]. We further aimed to involve large enterprises so as to have access to many workers at each work site, which facilitates logistics. The collaboration with large enterprises is of profound importance for solving logistic challenges with testing and measuring subjects in a field situation. This was a necessary decision even though it may give the study some lower representativeness toward smaller enterprises with possible differences in work environment. Within construction, subcontractors is often used by these large enterprises, which workers is assumed to possess a lot of the heaviest work tasks, are not investigated in this study. The study involves 1200 participants in total to secure a representative sample of employees and to provide the study with sufficient power to detect associations throughout the study. With this we aim to obtain a broad and representative description of exposures and outcomes by self-report for the 1200 and thereby for a variety of occupational titles within construction and health care. Since direct measurements are too resource-demanding to be carried out on all 1200 participants, we decided to establish the two subgroups of 300 and 160 in which objective exposure measurements were collected. Since all participants fill out the same questionnaire we can further determine if the subgroups differ significantly from the rest of the 1200 sample on several variables.

### Choosing objective exposure measurement

The choice of objective exposure measurement methods was a comprehensive part of planning this study. Our decision of using EMG, Accelerometers, heart rate and force insoles is an attempt to provide a profound basis of relevant objective exposure measures. We choose EMG since this is considered a good indicator of muscle work and studies using work shift EMG measurements have hypothesized muscle activity to be a factor affecting development of MSD [68, 83]. With our use of accelerometers, we are able to measure activity types and measure arm angles and upper body inclination. The technical possibilities in the Actigraph accelerometer also allowed us to include leisure time measurements for several days. The heart rate measurement by Actiheart was included to give a measure of the cardiovascular response during work and leisure. With this setup, we lacked a measure that quantified the external load during lifting exposure. To fill this need, pressure measurement insoles were introduced as an option and included in the measurements. This means that we can measure frequency and duration of lifting exposures during the work day. Our approach in exposure measurements leads to a notable amount of equipment to be carried by the subjects during measurement. The research group aim to obtain a good objective exposure measurement set-up without overloading participants, avoid hindering of their natural way of working and avoid possible loss of compliance due to this. To confront these challenges we carried out several pilot measurements, included both lab experiments and full-shift measurements in actual work conditions. Measurements were followed by conversations with workers on practicalities regarding carrying the measurement equipment during work tasks. Our solution with placing logger equipment in a specialized sports vest allows participants to carry this equipment at their back rather than in a belt at the hip, which is the alternative delivered by the manufacturer. This leads to minimal obstruction of work, especially advantageous for construction workers wearing a tool belt.

### Outcomes

Paper based questionnaire, smart phone questionnaire, register based sickness absence and physical examination will be repeated during a two-year follow-up period. The paper based questionnaire takes a wide range of variables into consideration and is therefore given to participants at six months interval too lower burden and thus minimize loss to follow-up. However, using intervals of six months may be too long to reflect fast fluctuations in health complaints and to capture recall of specific work events. Thus, the short monthly questionnaire sent by smart phone to the subgroups will cover these topics. Using national registers, we will be able to follow both the total and the diagnose-specific sickness absence. This will give a prospective time-line of the pattern of diagnosed sickness absence for the study population. The clinical examination undertaken in the subgroups was included to offer a detailed record of disorders in the musculoskeletal system at the end of the two year follow-up period.

### How to assess work ability

In addition to self-reported work ability we will also consider a measure of work ability in means of physical fitness relation to occupational demands. We use a sub-maximal test to estimate VO_2max_ instead of a maximal test that has a higher precision level than needed in this study, which is merely a crude classification of fitness status. Sub-maximal testing is further both time and equipment efficient. By using sub-maximal testing we also decrease the number of excluded participants since a larger number will have the needed qualifications to carry out a sub-maximal test compared to a maximal test. Thus we reduce loss of participants with health problems. The aerobic fitness test in combination with several days of measured heart rate further opens up for measuring work ability through e.g. percent of heart rate reserve [72]. Strength tests are fitted to a set of exercises acquiring minimal equipment in addition to be coordinated with establishment of EMG_max_.

Work ability through self reports is in this study measured by single-item rather than a comprehensive questionnaire, such as the Work Ability Index. Considering reports of good predictive value using the single item [57], this was chosen to be able to arrive at a manageable questionnaire, with reasonable compromise towards scientific strength.

### Study significance

A major strength of this study is the extensive investment in objectively measured biomechanical exposures, in a prospective design with repeated assessment of several relevant outcomes. To our knowledge, this is the first study to apply a comprehensive selection of instruments for mechanical exposure assessment in a real life working situation, including measurements of EMG, force, postures, physical activity and cardiovascular variables. We will further have rich information on self-reported mechanical exposures and possible confounders in this study, offering a good opportunity to adjust for confounding variables. With this study design we will obtain a profound basis of information that can be used to indicate associations between physical demands and musculoskeletal disorders, work ability and sickness absence.

## Abbreviations

MSD: Musculoskeletal disorders
ECG: Electrocardiography
EMG: Electromyography
MVC: Maximal voluntary contraction
Bpm: Beats per minute
VO_2max_: Maximal oxygen uptake
RMS: Root-mean-square.

## References

[1] Stephen B, Tatiana Q, Robin M, Michelle M, Anna V, Leela B (2009). Fit for Work? Musculoskeletal Disorders in the European Workforce.

[2] Miranda H, Kaila-Kangas L, Heliövaara M, Leino-Arjas P, Haukka E, Liira J, Viikari-Juntura E (2010). Musculoskeletal pain at multiple sites and its effects on work ability in a general working population. Occup Environ Med.

[3] van den Berg TIJ, Elders LAM, de Zwart BCH, Burdorf A (2009). The effects of work-related and individual factors on the work ability index: a systematic review. Occup Environ Med.

[4] Mantyselka PT, Kumpusalo EA, Ahonen RS, Takala JK (2002). Direct and indirect costs of managing patients with musculoskeletal pain-challenge for health care. Eur J Pain.

[5] da Costa BR, Vieira ER (2010). Risk factors for work-related musculoskeletal disorders: a systematic review of recent longitudinal studies. Am J Ind Med.

[6] Livshits G, Popham M, Malkin I, Sambrook PN, MacGregor AJ, Spector T, Williams FMK (2011). Lumbar disc degeneration and genetic factors are the main risk factors for low back pain in women: the UK Twin Spine Study. Ann Rheum Dis.

[7] Nilsen TIL, Holtermann A, Mork PJ (2011). Physical exercise, body mass index, and risk of chronic pain in the low back and neck/shoulders: longitudinal data from the nord-trondelag health study. Am J Epidemiol.

[8] Fujii T, Matsudaira K, Oka H (2013). Factors associated with fear-avoidance beliefs about low back pain. J Orthop Sci.

[9] National Research Council (2001). Musculoskeletal Disorders and the Workplace. Low Back and Upper Extremities.

[10] van Rijn RM, Huisstede BMA, Koes BW, Burdorf A (2010). Associations between work-related factors and specific disorders of the shoulder-a systematic review of the literature. Scand J Work Environ Health.

[11] Mayer J, Kraus T, Ochsmann E (2012). Longitudinal evidence for the association between work-related physical exposures and neck and/or shoulder complaints: a systematic review. Int Arch Occup Environ Health.

[12] Moriguchi CS, Carnaz L, Veiersted KB, Hanvold TN, Hæg LB, Hansson GÅ, Cote Gil Coury HJ (2013). Occupational posture exposure among construction electricians. Appl Ergon.

[13] Torgén M, Nygård CH, Kilbom Å (1995). Physical work load, physical capacity and strain among elderly female aides in home-care service. Eur J Appl Physiol.

[14] Engholm G, Holmström E (2005). Dose–response associations between musculoskeletal disorders and physical and psychosocial factors among construction workers. Scand J Work Environ Health.

[15] Skotte JH, Essendrop M, Hansen AF, Schibye B (2002). A dynamic 3D biomechanical evaluation of the load on the low back during different patient-handling tasks. J Biomech.

[16] Long MH, Bogossian FE, Johnston V (2013). The prevalence of work-related neck, shoulder, and upper back musculoskeletal disorders among midwives, nurses, and physicians: a systematic review. Workplace Health Saf.

[17] Videman T, Ojajarvi A, Riihimaki H, Troup JD (2005). Low back pain among nurses: a follow-up beginning at entry to the nursing school. Spine.

[18] Boschman JS, van der Molen HF, Sluiter JK, Frings-Dresen MHW (2012). Musculoskeletal disorders among construction workers: a one-year follow-up study. BMC Musculoskelet Disord.

[19] Stocks SJ, Turner S, McNamee R, Carder M, Hussey L, Agius RM (2011). Occupation and work-related ill-health in UK construction workers. Occup Med (Lond).

[20] Armstrong TJ, Buckle P, Fine LJ, Hagberg M, Jonsson B, Kilbom Å, Kuorinka IAA, Silverstein B, Sjøgaard G, Viikari-Juntura ERA (1993). A conceptual model for work-related neck and upper-limb musculoskeletal disorders. Scand J Work Environ Health.

[21] Ilmarinen J, Tuomi K, Eskelinen L, Nygård CH, Huuhtanen P, Klockars M (1991). Background and objectives of the Finnish research project on aging workers in municipal occupations. Scand J Work Environ Health.

[22] Alavinia SM, van Duivenbooden C, Burdorf A (2007). Influence of work-related factors and individual characteristics on work ability among Dutch construction workers. Scand J Work Environ Health.

[23] Ilmarinen JE (2001). Aging workers. Occup Environ Med.

[24] Sluiter JK (2006). High-demand jobs: age-related diversity in work ability?. Appl Ergon.

[25] Bern SH, Brauer C, Moller KL, Koblauch H, Thygesen LC, Simonsen EB, Alkjaer T, Bonde JP, Mikkelsen S (2013). Baggage handler seniority and musculoskeletal symptoms: is heavy lifting in awkward positions associated with the risk of pain?. BMJ Open.

[26] Silverstein M (2008). Meeting the challenges of an aging workforce. Am J Ind Med.

[27] Schibye B, Hansen AF, Søgaard K, Christensen H (2001). Aerobic power and muscle strength among young and elderly workers with and without physically demanding work tasks. Appl Ergon.

[28] Torgén M, Punnett L, Alfredsson L, Kilbom Å (1999). Physical capacity in relation to present and past physical load at work: a study of 484 men and women aged 41 to 58 years. Am J Ind Med.

[29] Russo A, Onder G, Cesari M, Zamboni V, Barillaro C, Capoluongo E, Pahor M, Bernabei R, Landi F, Ferrucci L (2006). Lifetime occupation and physical function: a prospective cohort study on persons aged 80 years and older living in a community. Occup Environ Med.

[30] Cassou B, Derriennic F, Iwatsubo Y, Amphoux M (1992). Physical diability after retirement and occupational risk factors during working life: a cross sectional epidemiological study in the Paris area. J Epidemiol Community Health.

[31] Li CY, Wu SC, Wen SW (2000). Longest held occupation in a lifetime and risk of disability in activities of daily living. Occup Environ Med.

[32] Holtermann A, Hansen JV, Burr H, Søgaard K, Sjøgaard G (2011). The health paradox of occupational and leisure-time physical activity. Br J Sports Med.

[33] Hubscher M, Ferreira ML, Junqueira DR, Refshauge KM, Maher CG, Hopper JL, Ferreira PH (2014). Heavy domestic, but not recreational, physical activity is associated with low back pain: Australian Twin low BACK pain (AUTBACK) study. Eur Spine J.

[34] Strijk JE, Proper KI, van Stralen MM, Wijngaard P, van Mechelen W, van der Beek AJ (2011). The role of work ability in the relationship between aerobic capacity and sick leave: a mediation analysis. Occup Environ Med.

[35] Arvidson E, Börjesson M, Ahlborg G, Lindegård A, Jonsdottir IH (2013). The level of leisure time physical activity is associated with work ability-a cross sectional and prospective study of health care workers. BMC Public Health.

[36] Gallagher S (2005). Physical limitations and musculoskeletal complaints associated with work in unusual or restricted postures: a literature review. J Safety Res.

[37] Takala EP, Pehkonen I, Forsman M, Hansson GÅ, Mathiassen SE, Neumann WP, Sjøgaard G, Veiersted KB, Westgaard RH, Winkel J (2010). Systematic evaluation of observational methods assessing biomechanical exposures at work. Scand J Work Environ Health.

[38] Kwak L, Proper KI, Hagströmer M, Sjöström M (2011). The repeatability and validity of questionnaires assessing occupational physical activity - a systematic review. Scand J Work Environ Health.

[39] Roffey DM, Wai EK, Bishop P, Kwon BK, Dagenais S (2010). Causal assessment of occupational pushing or pulling and low back pain: results of a systematic review. Spine J.

[40] Roffey DM, Wai EK, Bishop P, Kwon BK, Dagenais S (2010). Causal assessment of workplace manual handling or assisting patients and low back pain: results of a systematic review. Spine J.

[41] Wai EK, Roffey DM, Bishop P, Kwon BK, Dagenais S (2010). Causal assessment of occupational lifting and low back pain: results of a systematic review. Spine J.

[42] Roffey DM, Wai EK, Bishop P, Kwon BK, Dagenais S (2010). Causal assessment of occupational standing or walking and low back pain: results of a systematic review. Spine J.

[43] Roffey DM, Wai EK, Bishop P, Kwon BK, Dagenais S (2010). Causal assessment of occupational sitting and low back pain: results of a systematic review. Spine J.

[44] Roffey DM, Wai EK, Bishop P, Kwon BK, Dagenais S (2010). Causal assessment of awkward occupational postures and low back pain: results of a systematic review. Spine J.

[45] Wai EK, Roffey DM, Bishop P, Kwon BK, Dagenais S (2010). Causal assessment of occupational bending or twisting and low back pain: results of a systematic review. Spine J.

[46] Takala EP, Viikari-Juntura E, Moneta GB, Saarenmaa K, Kaivanto K (1992). Seasonal variation in neck and shoulder symptoms. Scand J Work Environ Health.

[47] Miranda H, Gold JE, Gore R, Punnett L (2006). Recall of prior musculoskeletal pain. Scand J Work Environ Health.

[48] Dallner M, Elo AL, Gamberale F, Hottinen V, Knardahl S, Lindström K, Skogstad A, Orhede E (2000). Validation of the General Nordic Questionnaire (QPS_Nordic_) for psychological and social factors at work. Nordic Council of Ministers, Nord.

[49] Statistics, Norway (2009). Levekårsundersøkelsen 2009 (Survey of level of living 2009).

[50] Eriksen W, Bjorvatn B, Bruusgaard D, Knardahl S (2008). Work factors as predictors of poor sleep in nurses’ aides. Int Arch Occup Environ Health.

[51] Noble BJ, Borg GAV, Jacobs I, Ceci R, Kaiser P (1983). A category-ratio perceived exertion scale: relationship to blood and muscle lactates and heart rate. Med Sci Sports Exerc.

[52] Saltin B, Grimby G (1968). Physiological analysis of middle-aged and old former athletes. Comparison with still active athletes of the same ages. Circulation.

[53] Steingrímsdóttir ÓA, Vøllestad NK, Røe C, Knardahl S (2004). Variation in reporting of pain and other subjective health complaints in a working population and limitations of single sample measurements. Pain.

[54] Theorell T, Harms-Ringdahl K, Ahlberg-Hultén G, Westin B (1991). Psychosocial job factors and symptoms from the locomotor system - a multicausal analysis. Scand J Rehabil Med.

[55] Ware JE (2000). SF-36 health survey update. Spine (Phila Pa 1976).

[56] Pallesen S, Bjorvatn B, Nordhus IH, Sivertsen B, Hjornevik M, Morin CM (2008). A new scale for measuring insomnia: the Bergen Insomnia Scale. Percept Mot Skills.

[57] Ahlstrom L, Grimby-Ekman A, Hagberg M, Dellve L (2010). The work ability index and single-item question: associations with sick leave, symptoms, and health - a prospective study of women on long-term sick leave. Scand J Work Environ Health.

[58] Astrand PO, Rodahl K, Dahl HA, Stromme BS (2003). Evaluation of Physical Performance on the Basis of Tests. Textbook of Work Physiology.

[59] Åstrand PO, Ryhming I (1954). A nomogram for calculation of aerobic capacity (physical fitness) from pulse rate during sub-maximal work. J Appl Physiol.

[60] Åstrand I (1960). Aerobic work capacity in men and women with special reference to age. Acta Physiol Scand Suppl.

[61] Essendrop M, Schibye B, Hansen K (2001). Reliability of isometric muscle strength tests for the trunk, hands and shoulders. Int J Ind Ergon.

[62] Biering-Sørensen F (1984). Physical measurements as risk indicators for low back trouble over a one-year period. Spine.

[63] Sluiter JK, Rest KM, Frings-Dresen MHW (2001). Criteria document for evaluating the work-relatedness of upper-extremity musculoskeletal disorders. Scand J Work Environ Health.

[64] Lærum E, Brox JI, Storheim K, Espeland A, Haldorsen E, Munch-Ellingsen J, Nielsen LL, Rossvoll I, Skouen JS, Stig LC, Werner EL (2007). Nasjonale Kliniske Retningslinjer. Korsryggsmerter – med og Uten Nerverotaffeksjon. (National Clinical Guidelines. Low Back Pain - With and Without Nerve Root Involvement).

[65] Mathiassen SE, Winkel J, Hägg GM (1995). Normalization of surface EMG amplitude from the upper trapezius muscle in ergonomic studies - a review. J Electromyogr Kinesiol.

[66] Hansson GÅ, Asterland P, Kellerman M (2003). Modular data logger system for physical workload measurements. Ergonomics.

[67] Hermens H, Freriks B, Merletti R, Stegeman D, Blok J, Rau G, Disselhorst-Klug C, Hägg G (1999). SENIAM 8: European Recommendations for Surface Electromyography.

[68] Cram JR (2003). The history of surface electromyography. Appl Psychophysiol Biofeedback.

[69] Brage S, Brage N, Ekelund U, Luan J, Franks PW, Froberg K, Wareham NJ (2006). Effect of combined movement and heart rate monitor placement on physical activity estimates during treadmill locomotion and free-living. Eur J Appl Physiol.

[70] Brage S, Brage N, Franks PW, Ekelund U, Wareham NJ (2005). Reliability and validity of the combined heart rate and movement sensor Actiheart. Eur J Clin Nutr.

[71] Kristiansen J, Korshøj M, Skotte JH, Jespersen T, Søgaard K, Mortensen OS, Holtermann A (2011). Comparison of two systems for long-term heart rate variability monitoring in free-living conditions-a pilot study. Biomed Eng Online.

[72] Gupta N, Jensen BS, Sogaard K, Carneiro IG, Christiansen CS, Hanisch C, Holtermann A (2014). Face validity of the single work ability item: comparison with objectively measured heart rate reserve over several days. Int J Environ Res Public Health.

[73] Redfern M, Hughes R, Chaffin D (1993). High-pass filtering to remove electrocardiographic interference from torso EMG recordings. Clin Biomech (Bristol, Avon).

[74] Drake JD, Callaghan JP (2006). Elimination of electrocardiogram contamination from electromyogram signals: An evaluation of currently used removal techniques. J Electromyogr Kinesiol.

[75] Jonsson B (1982). Measurement and evaluation of local muscular strain in the shoulder during constrained work. J Hum Ergol.

[76] Veiersted KB, Forsman M, Hansson GÅ, Mathiassen SE (2013). Assessment of time patterns of activity and rest in full-shift recordings of trapezius muscle activity - Effects of the data processing procedure. J Electromyogr Kinesiol.

[77] Østensvik T, Veiersted KB, Nilsen P (2009). A method to quantify frequency and duration of sustained low-level muscle activity as a risk factor for musculoskeletal discomfort. J Electromyogr Kinesiol.

[78] Skotte J, Korshøj M, Kristiansen J, Hanisch C, Holtermann A (2014). Detection of physical activity types using triaxial accelerometers. J Phys Act Health.

[79] Korshøj M, Skotte JH, Christiansen CS, Mortensen P, Kristiansen J, Hanisch C, Ingebrigtsen J, Holtermann A (2014). Validity of the Acti4 software using ActiGraph GT3X + accelerometer for recording of arm and upper body inclination in simulated work tasks. Ergonomics.

[80] Jebens E, Medbø JI, Knutsen O, Mamen A, Veiersted KB (2014). Association between perceived present working conditions and demands versus attitude to early retirement among construction workers. Work.

[81] Sterne JAC, White IR, Carlin JB, Spratt M, Royston P, Kenward MG, Wood AM, Carpenter JR (2009). Multiple imputation for missing data in epidemiological and clinical research: potential and pitfalls. Br Med J.

[82] Statistics, Norway (2014). Labour Force Survey.

[83] Hanvold TN, Wærsted M, Mengshoel AM, Bjertness E, Stigum H, Twisk J, Veiersted KB (2013). The effect of work-related sustained trapezius muscle activity on the development of neck and shoulder pain among young adults. Scand J Work Environ Health.

[CR84] The pre-publication history for this paper can be accessed here:http://www.biomedcentral.com/1471-2458/14/1075/prepub

